# WRKYs, the Jack-of-various-Trades, Modulate Dehydration Stress in *Populus davidiana*—A Transcriptomic Approach

**DOI:** 10.3390/ijms20020414

**Published:** 2019-01-18

**Authors:** Qari Muhammad Imran, Sang-Uk Lee, Bong-Gyu Mun, Adil Hussain, Sajjad Asaf, In-Jung Lee, Byung-Wook Yun

**Affiliations:** 1 Laboratory of Plant Functional Genomics, School of Applied Biosciences, Kyungpook National University, Daegu 41566, Korea; mimranbot@gmail.com (Q.M.I.); uk0gam@gmail.com (S.-U.L.); mun0301@naver.com (B.-G.M.); 2 Department of Agriculture, Abdul Wali Khan University, Mardan 23200, Pakistan; adilhussain@awkum.edu.pk; 3 Natural and Medical Sciences Research Center, University of Nizwa, Nizwa 616, Oman; sajadasif2000@gmail.com; 4 Laboratory of Plant Physiology, School of Applied Biosciences, Kyungpook National University, Daegu 41566, Korea; ijlee@knu.ac.kr

**Keywords:** RNA-seq analysis, *Populus davidiana*, WRKY transcription factor, dehydration stress, stomatal regulation

## Abstract

*Populus davidiana*, native to Korea and central Asian countries, is a major contributor to the Korean forest cover. In the current study, using high-throughput RNA-seq mediated transcriptome analysis, we identified about 87 *P. davidiana* WRKY transcription factors (PopdaWRKY TFs) that showed differential expression to dehydration stress in both sensitive and tolerant cultivars. Our results suggested that, on average, most of the WRKY genes were upregulated in tolerant cultivars but downregulated in sensitive cultivars. Based on protein sequence alignment, *P. davidiana* WRKYs were classified into three major groups, I, II, III, and further subgroups. Phylogenetic analysis showed that WRKY TFs and their orthologs in Arabidopsis and rice were clustered together in the same subgroups, suggesting similar functions across species. Significant correlation was found among qRT-PCR and RNA-seq analysis. In vivo analysis using model plant Arabidopsis showed that *atwrky62* (orthologous to Potri.016G137900) knockout mutants were significantly sensitive to dehydration possibly due to an inability to close their stomata under dehydration conditions. In addition, a concomitant decrease in expression of ABA biosynthetic genes was observed. The *AtHK1* that regulates stomatal movement was also downregulated in *atwrky62* compared to the wild type. Taken together, our findings suggest a regulatory role of PopdaWRKYs under dehydration stress.

## 1. Introduction

Plants, in their natural ecosystem, face several biotic and abiotic adversities. For their survival, plants respond to these adverse conditions through a variety of adaptive measures that include a series of complex pathways fine-tuned to regulate cellular processes necessary for coping with the adverse conditions. Water deficiency or drought is one of the major environmental constraints that disturb cellular homeostasis, leading to significant reduction in production throughout the world [1]. Drought influences a wide range of physiological processes in plants [2]. These include reduction in photosynthesis [3,4], stomatal closure [5,6], and wilting.

The production of reactive oxygen species (ROSs) is a common phenomenon under drought stress conditions. ROSs interact with several cellular signaling pathways and, when produced in high quantities, destroy lipids, thereby causing membrane injury [7]. The impact of drought stress on various plant parts, including leaf, stem, and floral parts, has been well-studied [8]. Recently, Gewin [9] demonstrated the downstream effects of drought stress on root architecture. Disturbance in root architecture decreases the development of lateral roots [10]. Degradation of lateral roots is associated with increased hydrotropism in the primary root [11]. This can further result in phytohormonal imbalance in plants. Changes in key plant hormones, such as auxin, cytokinin, gibberellin, and abscisic acid (ABA), during root responses to drought stress have been established [8,12]. Thus, complex signaling cascades contribute to the response of plants to drought stress [13]. These responses are mediated by changes in gene expression in different plant species, such as rice [14], maize [15], and pine [16].

Several studies have indicated that these regulatory mechanisms are mainly controlled via transcriptional activation or repression of specific regulatory genes [17,18,19]. Environmental stresses often arise in combination or in succession. Hence, survival in a variable environment involves multiple mechanisms. Therefore, it is important to study the molecular mechanisms linked with signal transduction leading to alteration of gene expression, in order to combat environmental stresses. The regulation of gene expression is carried out by a specific group of genes called transcription factors (TFs). The WRKY TF family is one of the largest and most important plant-specific TF families. Members of this family are largely involved in the modulation of various biological processes, such as nutrient deficiency, seed development, senescence [20], response to pathogens [21], growth and development [22], and most importantly, response to salt and drought stress [23,24]. In a recent study, Imran et al. [25] have identified 33 WRKY TFs that were differentially regulated in response to nitric oxide donor S-nitrosocysteine (CySNO) in an RNA-seq-based transcriptomic study. Several WRKYs have been identified in different plant species, including 72 in Arabidopsis (*Arabidopsis thaliana*) [26], 68 in sorghum (*Sorghum bicolor*), 38 in spreading earth moss (*Physcomitrella patens*) [27], 35 in spike moss (*Selaginella*) [28], 80 in pine (*Pinus monticola*) [29], approximately 45 in barley (*Hordeum vulgare*) [30], and more than 100 in rice (*Oryza sativa*) and soybean (*Glycine max*) [31,32,33]. Members of this family are characterized by the presence of either one or two copies of a specific hepta-peptide sequence, WRKYGQK, followed by an N-terminus zinc-finger motif, i.e., Cys(2)-His(2) (C2H2) or Cys(2)-HisCys (C2HC) [28]. The hepta-peptide sequence (WRKYGQK) specifically binds to consensus cis-acting elements (i.e., W-box, sequence = TTGACT/C) in the promoter region of target genes, thus regulating their transcription [28]. The number of WRKY domain(s) and pattern of the zinc-finger motif helps in the classification of WRKYs into different groups and subgroups [26,28]. The Group I WRKYs typically have double WRKY domains, whereas Group II members have a single. Group II WRKY members can be further subdivided, based on the pattern of the zinc-finger motif into IIa (C-X5-CX23-HXH), IIb (C-X5-CX23-HXH), IIc (C-X4-CX23-HXH), IId (C-X5-CX23-HXH), and IIe (C-S5-CX23-HXH). A single WRKY domain and a C2HC zinc-finger motif characterize Group III WRKY TFs. Various reports suggest that only the C-terminal WRKY domain can bind with DNA, whereas the N-terminal WRKY cannot, instead it helps the C-terminus WRKY in binding with DNA [28].

Trees, being the primary producers, play a crucial role in maintaining ecological balance. A forest cover of at least 25% of the total geographic area of a particular region is mandatory to maintain this balance in that area. Among the several reasons for the continuously decreasing global forest cover, water shortage, or drought is one of the most important [34,35]. Populus, a genus comprised of about 35 different species of deciduous flowering plants, is native to the northern hemisphere. The western balsam poplar or black cottonwood (*P. trichocarpa*) was the first tree species for which a complete genome was sequenced in 2006 [36]. *P. davidiana*, native to the Korean peninsula, has evolved to overcome various environmental stresses. It is used for multiple purposes and constitutes a major part of the forest cover in Korea. However, the changing environmental conditions, specifically drought, pose a major challenge to these forest trees’ survival. According to an estimate, the Republic of Korea lost 1.7% of its forest cover during the period from 1990 to 2005, which is equivalent to an area of around 106,000 hectares (http://rainforests.mongabay.com/deforestation/archive/South_Korea.htm#ref). Extensive reports reveal that the genus *Populus* is a favorable candidate for research on forest trees under stress conditions [37,38,39]. However, with the exception of *P. trichocarpa*, no genomic information is available for other poplar species so far. In this study, we present the first transcriptome-wide identification and characterization of WRKY TFs in *P. davidiana* (Korean aspen) in response to dehydration stress. This study will provide useful information about global gene expression in response to dehydration stress and will help in understanding the molecular mechanism underlying drought stress tolerance in *P. davidiana* and other forest trees.

## 2. Results

### 2.1. Selection of Drought Sensitive and Tolerant Cultivars

**Figure 1:**
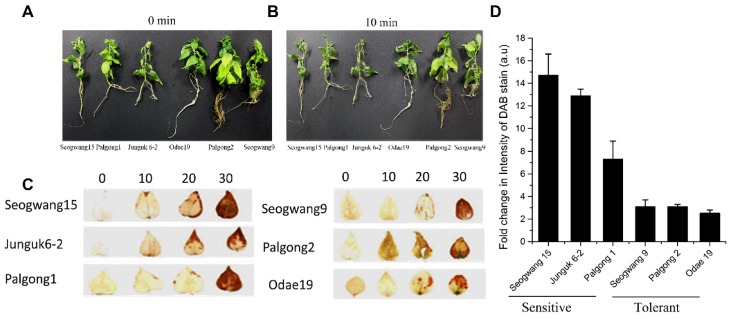
Exposure of *P. davidiana* plants to dehydration stress: (**A**) Different *P. davidiana* cultivars before and (**B**) after 10 min of air-drying; (**C**) DAB staining as a measure of H${}_{2}$O${}_{2}$ accumulation in the indicated *P. davidiana* cultivars; (**D**) Fold change of intensity of the DAB staining measured through the Luminosity tool in Photoshop by fixing the values for the rectangular marquee tool. All the data points are the mean of three replicates and error bars represents ± SE.

About 22 different *P. davidiana* cultivars were screened for sensitivity or tolerance toward dehydration stress based on symptoms development and H${}_{2}$O${}_{2}$ accumulation. Our results suggested that Seogwang15 was the most sensitive cultivar having highest accumulation of H${}_{2}$O${}_{2}$ after 20 and 30 min of stress followed by Junguk6-2 and Palgong1 (Figure 1A–D). Among tolerant cultivars, H${}_{2}$O${}_{2}$ accumulation started after 20 min of stress except Palgong2 that started H${}_{2}$O${}_{2}$ accumulation after 10 min; however, the plants were still healthy (Figure 1C). After keen observation, three representative sensitive and tolerant cultivars were selected for RNA-seq analysis.

### 2.2. Transcriptome-Wide Identification of PopdaWRKYs

**Figure 2:**
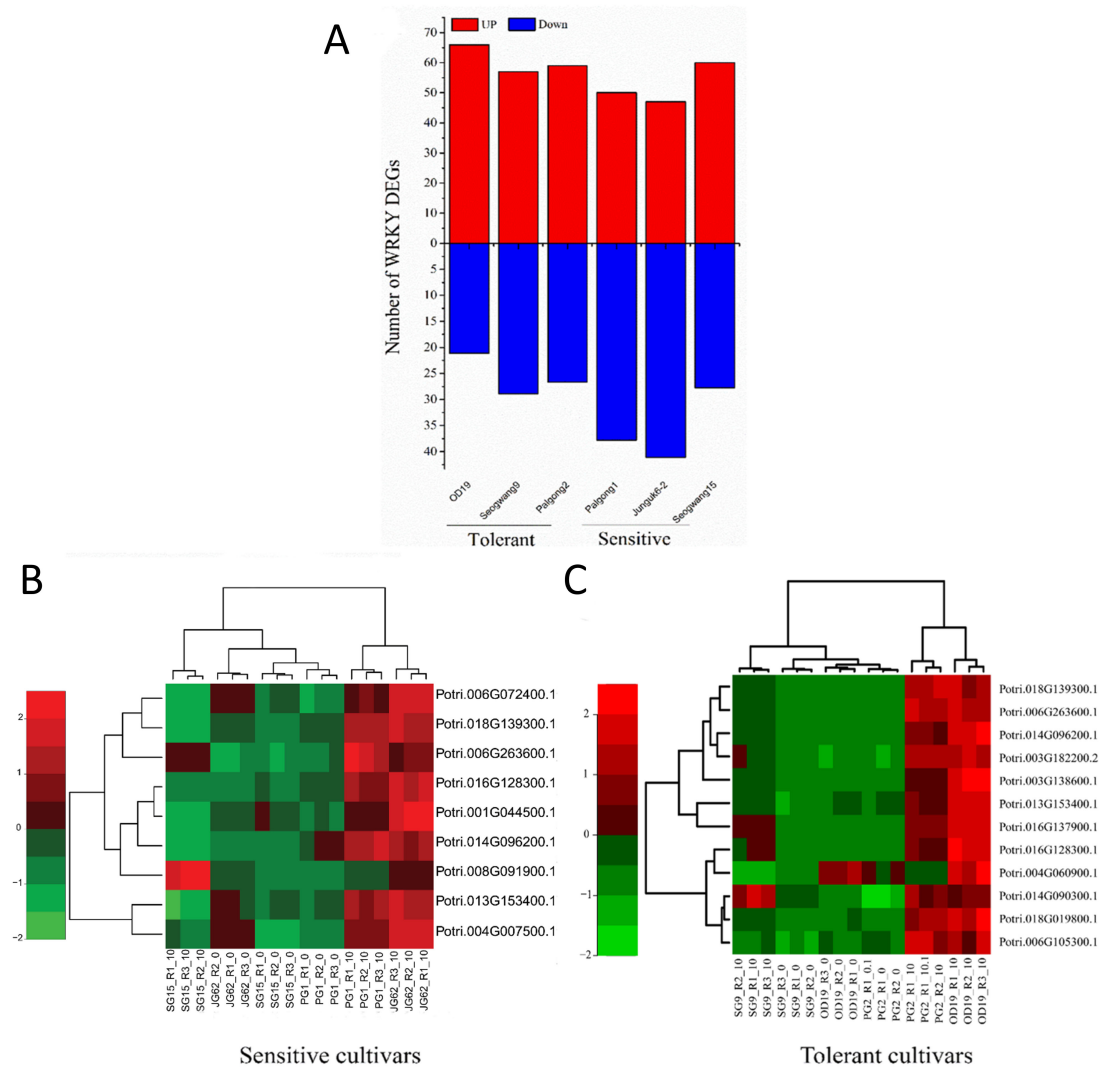
Drought-induced WRKY DEGs in sensitive and tolerant *P. davidiana* cultivars: (**A**) Total number of WRKY DEGs (upregulated and downregulated) in all sensitive and tolerant cultivars. (**B**) Heatmap showing expression patterns with dendrogram representing hierarchical clustering of common DEGs in sensitive and (**C**) tolerant *P. davidiana* cultivars. The heatmaps were generated from the significant WRKY DEGS (p≤0.05) using FPKM values in three replicates using *R*.

We identified a total of 22,522 DEGs (sum of DEGs in all cultivars) in response to dehydration stress in both sensitive and tolerant *P. davidiana* cultivars. We further sought to identify and characterize dehydration-responsive PopdaWRKY TF genes. A total of 87 PopdaWRKY TFs (including non-significant DEGs) were identified in both sensitive and tolerant *P. davidiana* cultivars. However, the number of upregulated and downregulated WRKYs in sensitive and tolerant cultivars varies, with sensitive cultivars having more downregulated WRKY TF genes and tolerant cultivars having more upregulated WRKY genes (Figure 2A). In tolerant cultivars (OD19, Seogwang9, and Palgong2), the numbers of upregulated WRKY TF genes were 66, 57, and 59, respectively, while the number, of downregulated genes were 19, 26, and 24, respectively. Similarly, in sensitive cultivars (Palgong1, Junguk6-2, and Seogwang15), the numbers of upregulated WRKY TFs were 50, 47, and 60, while the numbers of downregulated WRKY genes were 34, 37, and 25 (Figure 2A). Heatmaps of PopdaWRKY DEGs in both sensitive and tolerant cultivars were generated from the respective FPKM values, showing hierarchical clustering and gene expression intensities (Figure S1). We further selected significant (p≤0.05) DEGs in both sensitive and tolerant cultivars and generated a heatmap with a dendrogram showing hierarchical clustering to see the difference in their expression level in both (sensitive and tolerant) cultivars (Figure 2B,C). We observed that Potri.018G139300.1, Potri.006G263600.1, Potri.016G128300.1, Potri.014G096200.1, and Potri.013G153400.1 were common in both sensitive and tolerant cultivars. Interestingly, all these common genes were upregulated in both cultivars (Figure 2B,C). MDS plot showing dispersion among data is also presented in Figure S2.

### 2.3. Global Gene Regulation Is Different in Sensitive and Tolerant *P. davidiana* Cultivars

To identify the WRKY TFs common to both sensitive and tolerant *P. davidiana* cultivars, we examined WRKY DEGs from both (sensitive and tolerant) cultivars. The DEGs from all these cultivars were analyzed using online tool by VIB-UGENT (http://bioinformatics.psb.ugent.be/webtools/Venn/). Our results suggested five DEGs each were common between Junguk62, Odae19, Palgong1, Palgong2, Seogwang15 Seogwang9, respectively (Table 1). Three DEGs each were common between Junkguk62, Odae19 Palgong2 respectively (Table 1). The complete list of all common DEGs found among different cultivars is presented in Table 1. Although most of the DEGs were common among sensitive and tolerant cultivars, their global gene expression was different in both. For example, Potri.014G164300.1 and Potri.015G099200.1 was upregulated in tolerant cultivars but downregulated in sensitive cultivars, whereas Potri.001G121300.1 was downregulated in tolerant cultivars and upregulated in sensitive cultivars (Table S4).

### 2.4. Chromosomal Location of PopdaWRKYs and Their Orthologs in Other Species

**Figure 3:**
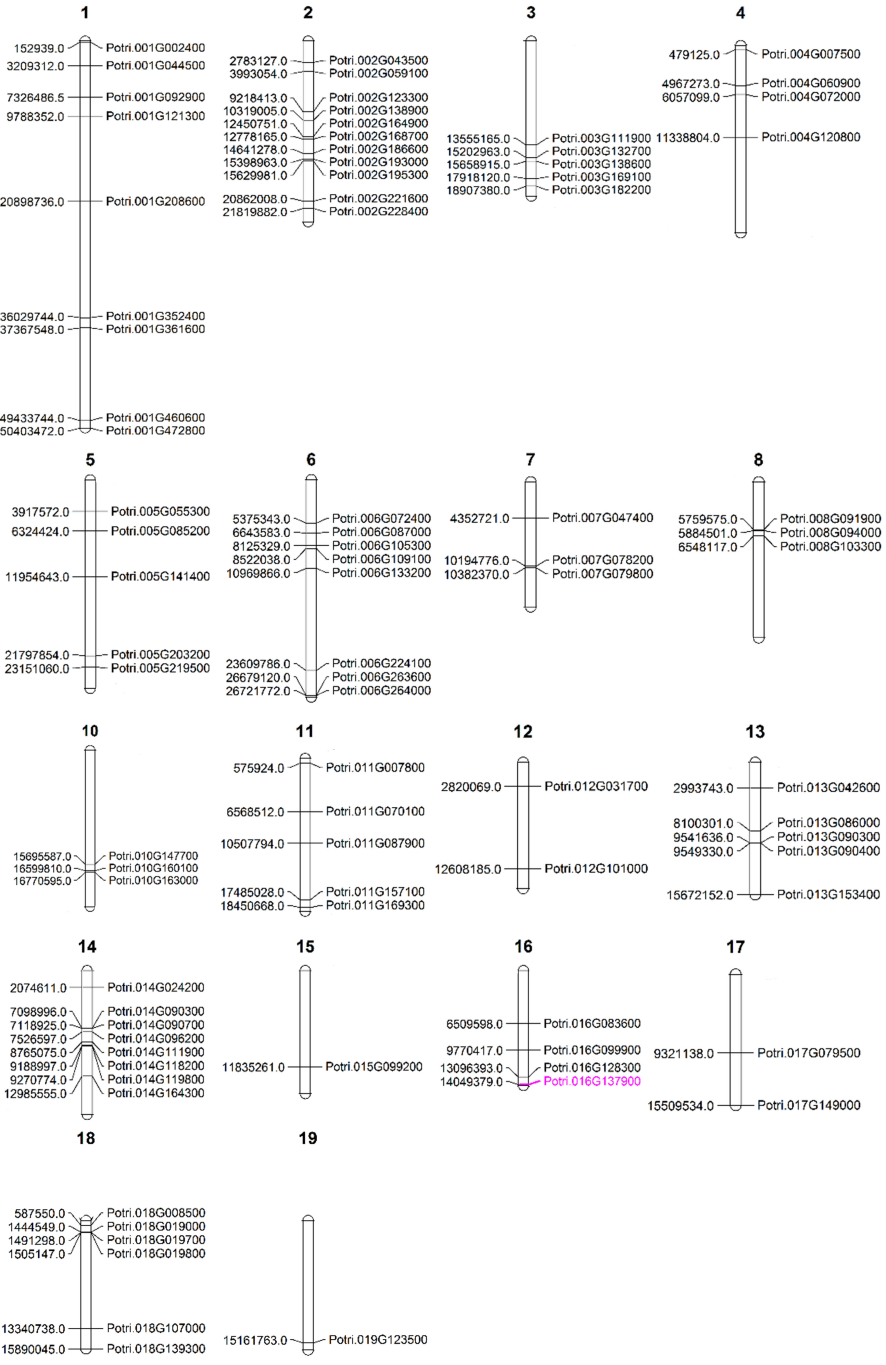
The predicted position of dehydration-responsive WRKYs on different chromosomes: The DEGs that were found common in all varieties at 10 min post stress were analyzed using Phytozome (https://phytozome.jgi.doe.gov/pz/portal.html) against the reference genome, and the position of these genes was then predicted on relative chromosomes using MapChart. The WRKY in purple is orthologous to Arabidopsis *AtWRKY62*, which was used later for in vivo analysis.

A gene’s location on chromosome determines the fate of a particular trait, as suggested by Rockman and Skrovanek [40], who stated that a gene’s location on the chromosome plays a key role in determining the variation and evolution of a particular trait in an organism. Therefore, we sought to determine the chromosomal locations of dehydration-responsive PopdaWRKYs. The majority of PopdaWRKYs were found on Chromosome 2, followed by 1 and 14, respectively, and the least were found on Chromosomes 15 and 19 (Figure 3). Interestingly, there was no dehydration-responsive WRKY associated with Chromosome 9. The predicted position of dehydration-responsive WRKYs on chromosomes was not uniform. For example, Chromosomes 2, 3, 6, 8, 10, 13, 14, and 18 are often found in clusters of at least three or more genes.

We also examined the PopdaWRKY orthologs in other species and found that they are distributed almost everywhere in the plant kingdom, including trees, field crops, vegetables, and grasses. The highest number of orthologs was found in *Manihot esculenta* and *Populus trichocarpa* (83 each), followed by *Anacardium occidentale* (cashew nut), *Theobroma cacao* (cocoa tree), *Prunus persica* (peach), *Ricinus communis* (castor bean), and *Salix purpurea* (purple osier willow) with 82, 79, 78, 76, and 76 orthologs, respectively (Table S2). The lowest number of PopdaWRKY orthologs were found in *Musa acuminata* (banana) and *Setaria viridis* (green bristlegrass) with 58 orthologs each (Table S2).

### 2.5. Fold Enrichment of GO Terms

To examine the putative function of PopdaWRKYs, fold enrichment of GO terms for biological processes and molecular functions were determined using PANTHER (release 20160715) overrepresentation tool, by selecting *P. trichocarpa* as the reference genome in the GO consortium database (http://geneontology.org/). From a total of 87 PopdaWRKYs, 80 were successfully mapped to the reference genome. Among GO terms for biological processes, regulation of transcription had the highest fold enrichment (16.69), followed by regulation of RNA biosynthetic process (16.43), regulation of RNA metabolic processes (16.3), and macromolecular biosynthetic process (15.9). GO terms for biological processes with *p* < 0.05 are presented in Table 2. The complete list of biological processes is also provided in Table S3. Similarly, GO terms for molecular functions revealed the highest fold enrichment for sequence-specific DNA binding (39.23), followed by transcription factor activity (33.46) (Table 2 and Table S3).

### 2.6. Classification and Phylogenetic Analysis

**Figure 4:**
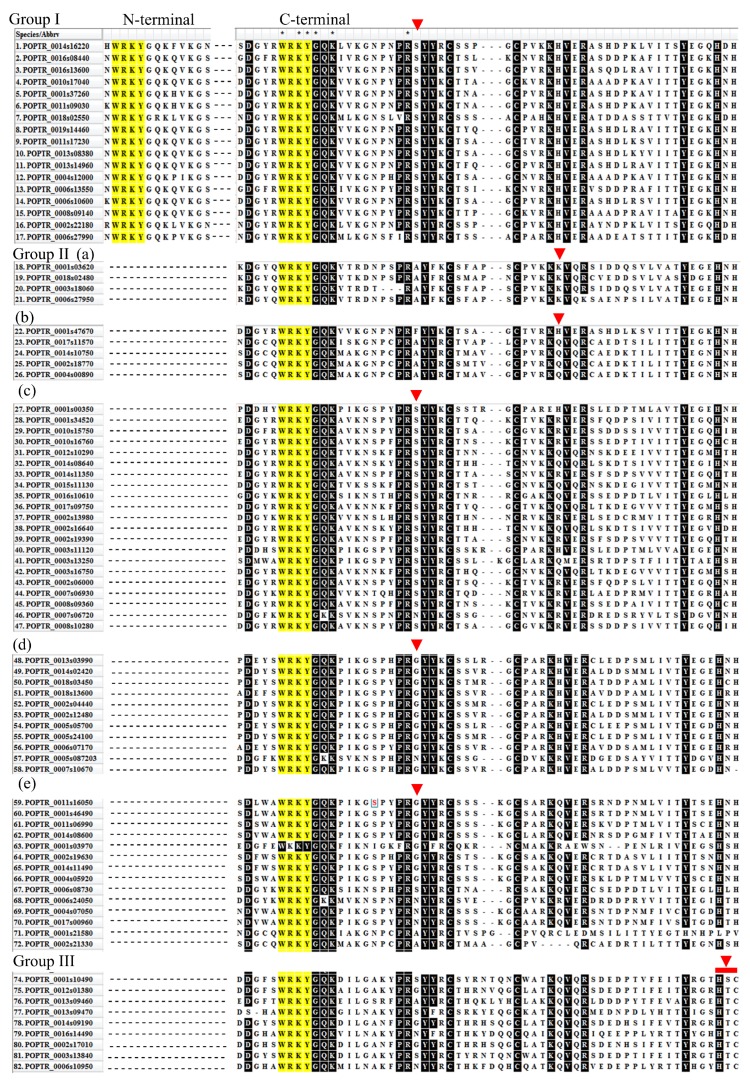
Protein sequence alignment of drought-induced WRKY TFs: All the sequences were aligned using ClustalW with default parameters in MEGA 7.0. Based on the number of WRKY domains, and the pattern of the zinc finger motif, these TFs were divided into different groups and sub-groups. Both N-terminal and C-terminal WRKY domains can be seen in Group I WRKYs, whereas Group II and III are characterized by only one WRKY domain, however, they differ by the C-terminal zinc finger motif composition. the rest have only a C-terminal WRKY domain. Only the WRKY domain has been highlighted here. Complete protein sequences are not shown. The black highlighted residues indicate conserved motifs/residues that were found in at least 90% of the sequences. Red triangles indicate a difference in motif composition from other groups. Dots represents missing protein sequences which were removed for optimal alignment and to show the conserved regions. The red rectangle in the down-most part represents the difference in the zinc finger motif of Group III and others.

The motif and domain composition of WRKY is important for understanding its involvement in a particular process. Therefore, we classified all dehydration-responsive PopdaWRKYs in different groups as mentioned by Rushton et al. [26]. PopdaWRKYs having both N- and C-terminal WRKY domains were grouped into Group I (Figure 4). We found about 17 Group I PopdaWRKYs distributed all over the genome (Figure 4). Group II members having a single C-terminal WRKY were further classified into five subgroups. Among a total of 55 Group II PopdaWRKY members, four were categorized in Group IIa, five in IIb, 21 in IIc, 11 in IId, and 14 in IIe (Figure 4). Similarly, nine members were categorized in Group III (Figure 4).

**Figure 5:**
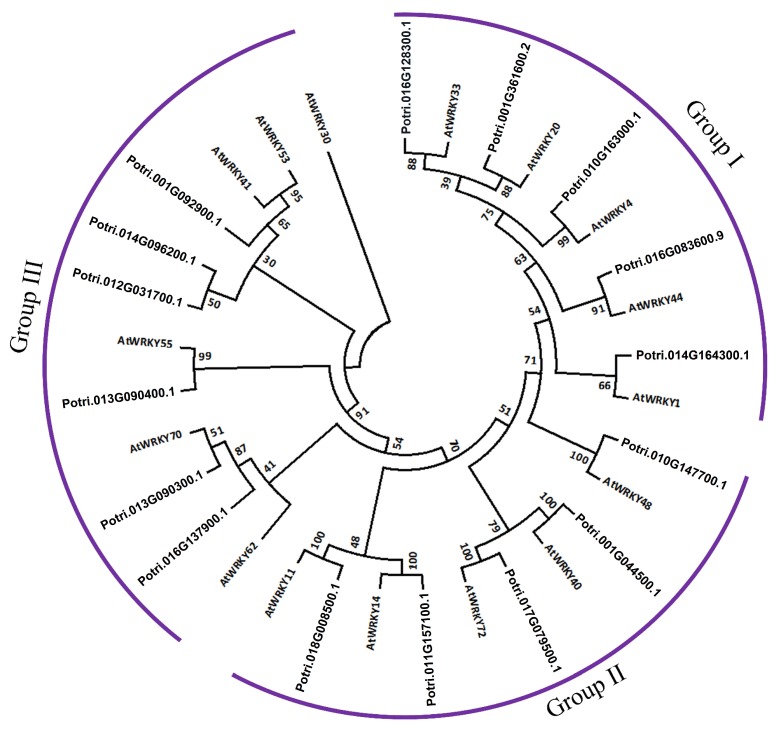
Phylogenetic analysis of PopdaWRKYs and their orthologs in Arabidopsis: The phylogenetic tree constructed from protein sequences of selected drought-induced PopdaWRKYs and their orthologs in Arabidopsis using the neighbor-joining method with a bootstrap value of 1000 replicates using Mega 7.0. All the WRKYs were grouped together within the same subgroup.

Although no genomic information is available for *P. davidiana*, the WRKY family has been extensively studied in Arabidopsis with all the relevant information available. We therefore sought to compare PopdaWRKYs with *A. thaliana* WRKYs (AtWRKYs) to confirm that they had been properly classified into different subgroups. Fifteen different PopdaWRKYs were selected, representing five each from Groups I, II, and III. The protein sequences of these TFs and their orthologs in Arabidopsis were aligned using MEGA7 [41]. The resultant alignment was used to construct a phylogenetic tree using the neighbor-joining method [42] with 1000 bootstrap replicates. All the WRKY TFs were classified into three major clades; Group I members from both species were grouped in a single clade, as was the case with Group II and III WRKY TFs (Figure 5). In Group I, the highest bootstrap values were recorded for AtWRKY4 and Potri.016G128300.1, followed by AtWRKY44 and Potri_016G083600.9. Similarly, in Group II, the highest bootstrap values were recorded for AtWRKY48, AtWRKY40, AtWRKY14, and AtWRKY11, and their corresponding orthologs in poplar. In the case of Group III WRKYs, the highest bootstrap value was recorded for AtWRKY55 and Potri_013G090400.1. AtWRKY62 was grouped together with Potri_016G137900.1.

### 2.7. Structural Divergence in Motif Composition of WRKYs in *P. davidiana* and Its Orthologs in Other Species

**Figure 6:**
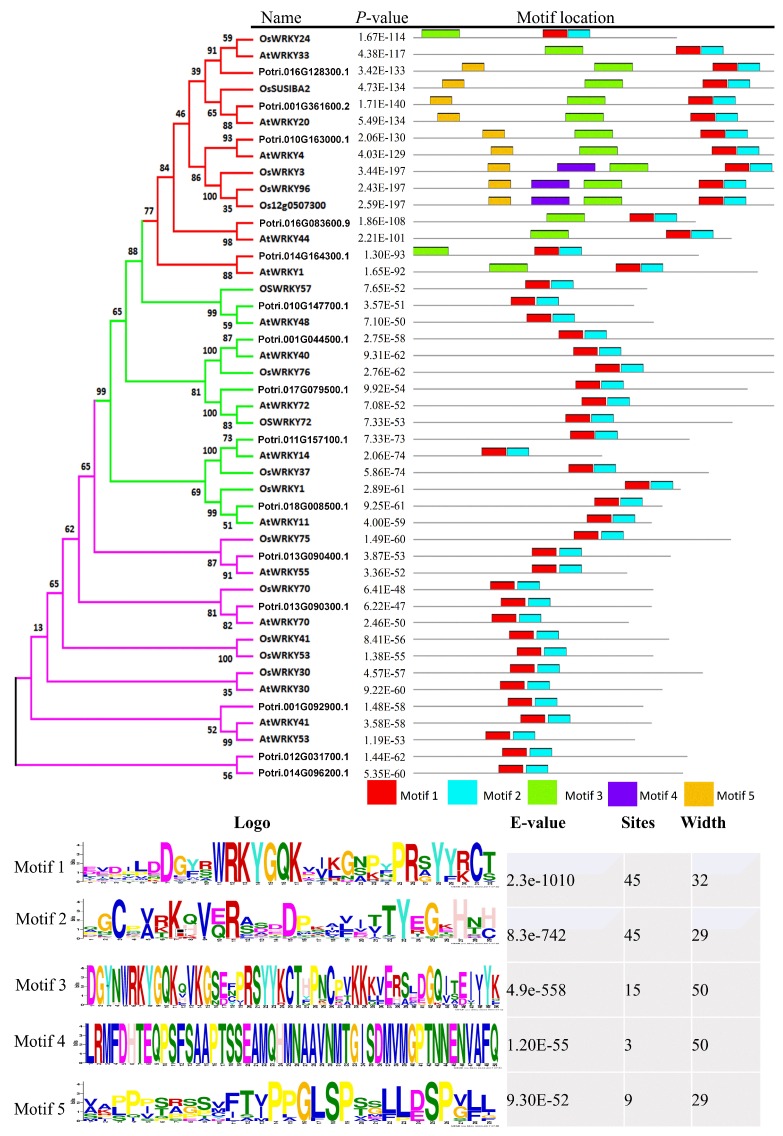
Phylogenetic relationship and motif composition of drought-induced WRKY TFs in *P. davidiana*: The phylogenetic tree shown was constructed using 45 WRKY (15 each from *P. davidiana*, Arabidopsis, and rice) protein sequences based on the neighbor-joining method. Evolutionary distances were computed using the Poisson correction method and are displayed as the number of amino acid substitutions per site. All positions containing gaps and missing data were eliminated. Evolutionary analysis was conducted using MEGA 7.0. Structural divergence in motif composition was conducted using MEME (http://meme-suite.org/tools/meme). A total of five motifs were studied. Motif 1 represents the C-terminal WRKY, and Motif 3 represents the N-terminal WRKY domain. The bottom part represents the detailed structure of the studied motifs.

To further understand the role of PopdaWRKYs in plant defense and stress tolerance, the dehydration-responsive PopdaWRKY TFs and their orthologs in Arabidopsis and rice were queried using the BLASTP option, against their respective reference genomes. All the protein sequences were aligned using MEGA7 [41], and a phylogenetic tree was constructed using the neighbor-joining method with 1000 bootstrap replicates. The conserved motifs were analyzed using the MEME tool (http://meme-suite.org/tools/meme). Our results showed that all the 45 WRKY members from different species grouped together in three major clades representing Group I, II, and III. Motifs 1 and 2 representing the C-terminal WRKY domains were conserved among all species, whereas Motif 3 representing the N-terminal WRKY domain was found only in Group I WRKY members (Figure 6). Furthermore, Motifs 4 and 5 were also found in some members of Group I WRKYs (Figure 6). Overall, a similar motif composition was found within the same group or subgroup of WRKY TFs from poplar, Arabidopsis, and rice, suggesting that these WRKY members perform similar functions in these plants.

### 2.8. Involvement of Dehydration-Responsive WRKYs in Different Metabolic Pathways

WRKY TFs regulate multiple cellular pathways. To examine the role of dehydration-responsive PopdaWRKYs in different pathways, dehydration-induced PopdaWRKYs were analyzed using the “Search and Color Pathway” tool in the Kyoto Encyclopedia of Genes and Genomes (KEGG) (http://www.genome.jp/kegg/pathway.html), on the basis of the reference pathway. The results indicated that dehydration-responsive WRKYs were putatively involved in two major pathways, the mitogen-activated protein kinase (MAPK) signaling pathway and the plant–pathogen interaction pathway. In the MAPK pathway, WRKY33 interacts with phytoalexin deficient3 (PAD3) that regulates camalexin synthesis (Figure S3). In a similar manner, but through a different route, WRKY22 interacts with FLG22-induced receptor-like kinase1 (FRK1) to mediate early defense responses, immediately after pathogen attack (Figure S3). WRKY22 is also involved in H${}_{2}$O${}_{2}$-mediated responses after pathogen attack (Figure S3). Similarly, in the plant–pathogen interaction pathway, WRKYs play a key role in conferring resistance to both bacterial and fungal pathogens. Following an attack by bacterial pathogens, WRKY22 and WRKY33 are induced through an unknown mechanism in both R-gene (resistance gene) mediated and Ca^2+^ signaling pathways, thus interacting with other genes to induce defense-related gene expression. Likewise, WRKY1 and WRKY2 are also involved in effector-triggered immunity in fungi (Figure S3).

### 2.9. RNA-seq and qRT-PCR Analysis Are Highly Correlated with Each Other

**Figure 7:**
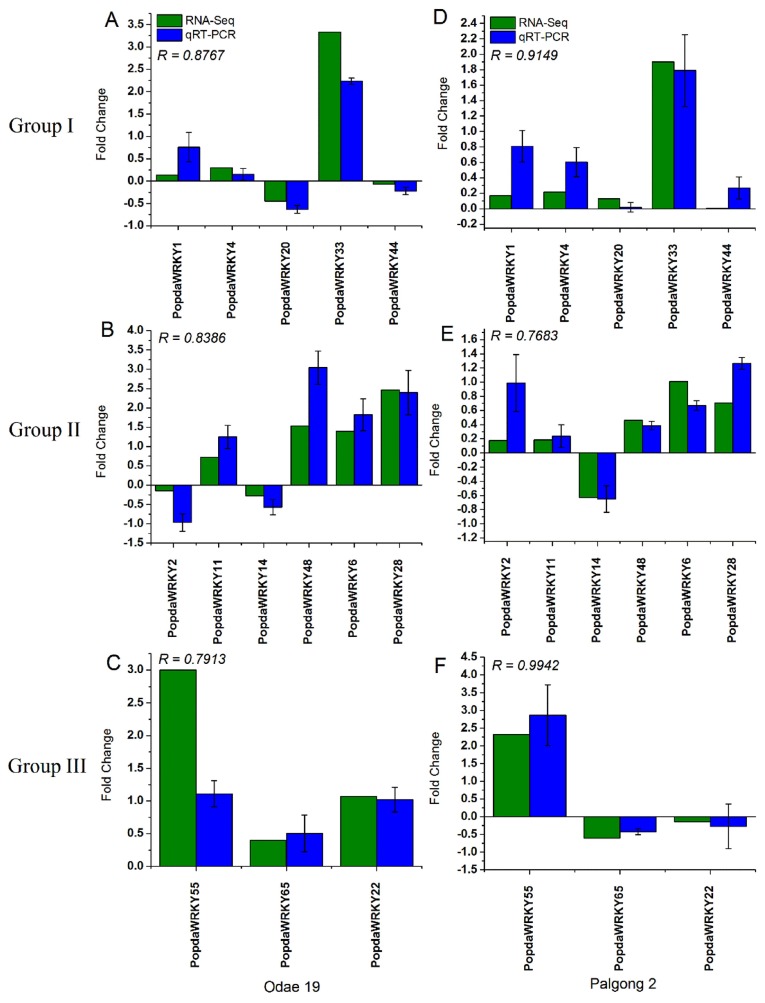
Validation of RNA-seq results using qRT-PCR in tolerant popular cultivars: The same 14 PopdaWRKYs were also studied in both tolerant cultivars, Odae 19 (**A**–**C**) and Palgong 2 (**D**–**F**). The fold change calculated from qRT-PCR analysis (white bars) was compared with fold change from RNA-seq analysis (black bars). The *R* value represents the correlation coefficient, while the error bars represent ± SE from at least three replicates.

To further confirm dehydration-induced transcriptional changes in PopdaWRKY TFs, 14 different PopdaWRKYs including five from Group I, six from Group II, and three from Group III were selected for qRT-PCR analysis in the top two tolerant and sensitive cultivars. The transcript accumulation of these genes was determined after 10 min of dehydration and compared with RNA-seq analysis. A high correlation coefficient (R < 6) indicates that both qRT-PCR and RNA-seq analysis are highly comparable. Our results suggested that, in both sensitive and tolerant popular cultivars, *PopdaWRKY33* showed the highest transcript accumulation compared to other members of the same group (Figure 7 and Figure S4). Similarly, *PopdaWRKY14* was the most downregulated gene among both cultivars compared to other members of the same group (Figure 7 and Figure S4). Among Group II WRKY members, *PopdaWRKY22* was downregulated in both sensitive cultivars and upregulated in both tolerant cultivars (Figure 7 and Figure S4). Similarly, *PopdaWRKY44* was downregulated in both sensitive cultivars and upregulated in one of the tolerant cultivar (Palgong 2) (Figure 7 and Figure S4). Among Group III WRKYs, *PopdaWRKY55* was upregulated in both tolerant cultivars but downregulated in the Seogwang15 sensitive cultivar (Figure 7 and Figure S4).

### 2.10. AtWRKY62 Modulates Dehydration Stress via Stomatal Regulation and Transcriptional Regulation of the ABA Pathway in *Arabidopsis thaliana*

**Figure 8:**
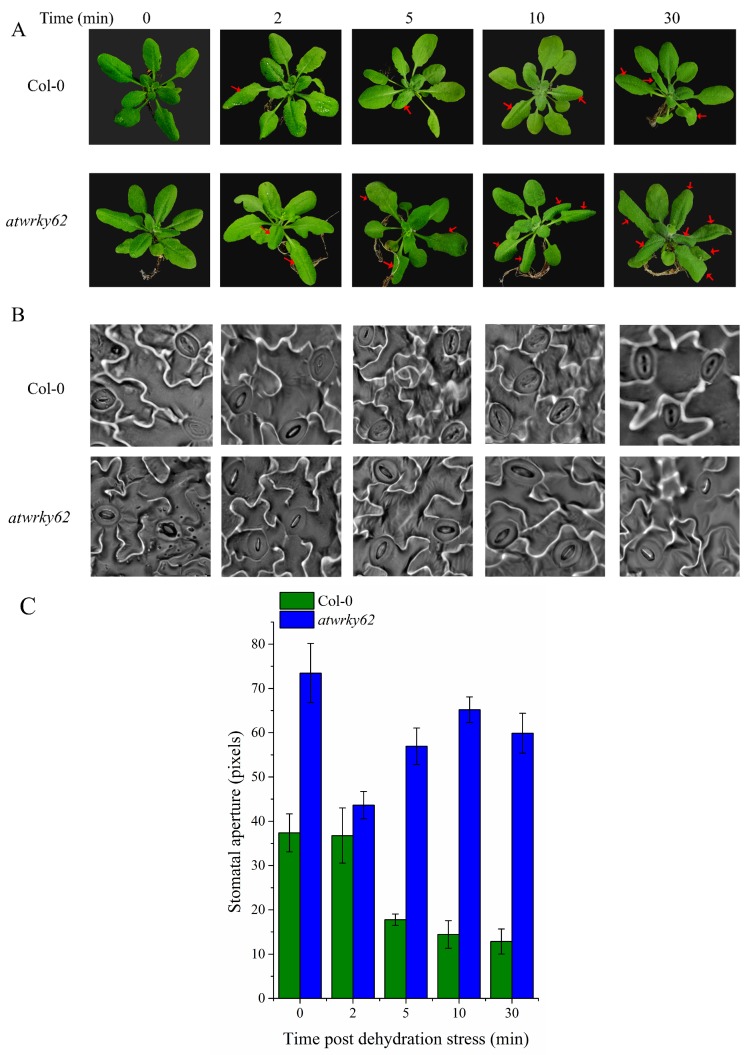
In vivo analysis of *AtWRKY62* after dehydration stress using functional genomics study: (**A**) Symptoms/wilting and downward curling of *atwrky62* and WT overtime after dehydration stress. The red arrows indicates the wilted and curled leaves post stress. (**B**) Stomatal conductance of indicated genotypes studied overtime after dehydration stress. (**C**) Stomatal aperture of indicated genotypes measured using the ruler tool in Adobe Photoshop CS6 in three replicates. Error bars represents ± SE.

In order to validate the regulatory role of WRKY TFs in vivo, Arabidopsis WT and *atwrky62* were exposed to dehydration stress as described in Section 2.9. Results suggested that WT plants did not show any signs of wilting, whereas *atwrky62* started wilting and downward curling of leaves even after 5 min (Figure 8A). To further investigate the sensitive response of *atwrky62* to dehydration, we performed microscopic observations of Col-0 and *atwrky62* leaves after dehydration stress. Results indicated that, after 2 min, WT plants showed the stomata, while in *atwrky62* stomata were clearly open even after 30 min of dehydration stress (Figure 8B). To quantify a stomatal opening, we measured the stomatal aperture using a ruler tool in Adobe Photoshop CS6. The result indicated that, at the 2 min time point, there was no significant difference between WT and *atwrky62* (Figure 8C). However, 5, 10, and 30 min post dehydration stress showed a significant difference in the stomatal aperture of WT and *atwrky62* (Figure 8C).

**Figure 9:**
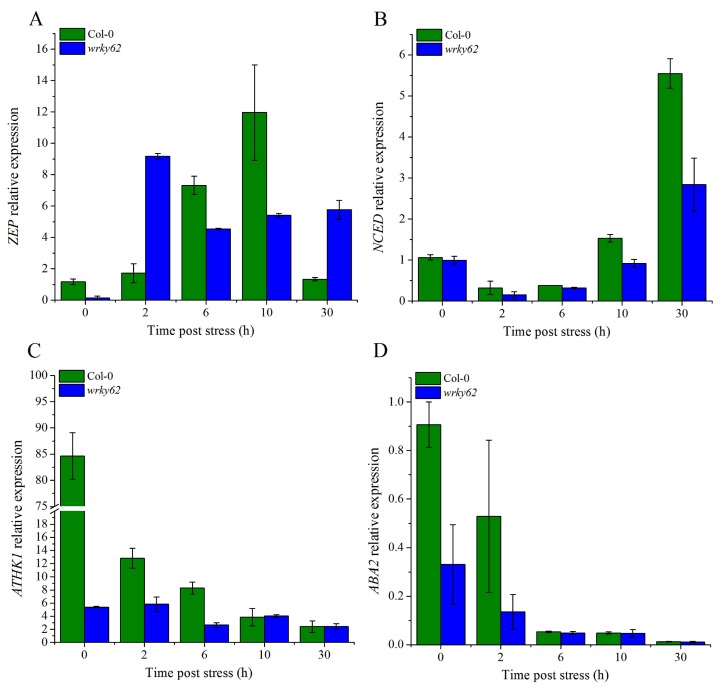
Relative expression of ABA biosynthesis/signaling genes: Transcript accumulation of (**A**) *ZEP*, (**B**) *NCED*, (**C**) *AtHK1*, and (**D**) *ABA2* after dehydration conditions in the indicated genotypes were analyzed using qRT-PCR. All the data points represents the mean of three replicates. Bars indicates ± standard error.

We were further interested in determining if this stomatal conductance was transcriptionally regulated. Therefore, we performed qRT-PCR to determine overtime changes in the expression of key genes involved in ABA biosynthesis and signaling [43,44,45,46] i.e., *ABA2*, *ZEP*, *NCED*, and *AtHK1*. Results suggested that *ZEP* showed reduced expression at 0, 6, and 10 min of dehydration (Figure 9A). Similarly, other ABA biosynthesis related genes such as *NCED* and *ABA2* also showed reduced expression compared to WT (Figure 9B,C). *AtHK1* also showed a reduced transcript accumulation in *atwrky62* compared to WT, at early time points (Figure 9 C).

## 3. Discussion

Drought stress is a universal problem, increasing at a rapid pace, especially in rain-fed areas with limited water reserves and poor irrigation practices. Trees, a major component of producers, function as thermal stabilizers in the ecosystem. However, they face both man-made and natural adversities, including drought. The increasing human population results in speedy deforestation globally, thus reducing the forest cover. Therefore, it is of prime importance to select high yielding breeds, which can tolerate multiple stress conditions, including drought. The appropriate selection of these cultivars requires a profound understanding of the underlying mechanisms that are mediated by drought stress in plants. *P. davidiana* (Korean aspen) is considered to be naturally drought tolerant. However, genomic information is not available for this tree. Global changes in gene expression are critical for understanding the molecular mechanisms of a particular organism. Poplar is considered to be a good candidate for research on forest trees, as suggested by Li et al. [47] and Qiu et al. [37]. WRKY TFs, also known as a “jack of all trades”, regulate various cellular processes, including response to drought and salinity stress [20,22]. Dehydration is considered to be a severe type of drought. Therefore, studying dehydration-induced drought-responsive WRKY TFs in *P. davidiana* can help in understanding the mechanistic control of global gene expression in response to drought stress.

In this study, we performed high-throughput RNA-seq mediated transcriptome analysis of drought sensitive and tolerant *P. davidiana* cultivars under dehydration stress conditions. We further identified WRKY TFs in both sensitive and tolerant cultivars, and discovered approximately 87 WRKYs that showed differential expression in both types of cultivars under dehydration stress (Table S4). It is noteworthy that the number of upregulated and downregulated genes varies in sensitive and tolerant cultivars. Overall, the tolerant group has more upregulated WRKY DEGs, whereas the sensitive group possesses more downregulated WRKY DEGs. This could be because of a possible stress tolerance strategy, as reported by Muthusamy et al. [48], which suggests that the large number of downregulated genes in sensitive banana cultivars is either the direct negative impact of the stress or a part of the stress adaptation strategy. This prompted us to study the common and unique genes between the sensitive and tolerant cultivars. Our results showed that the majority (90.8%) of WRKY DEGs were found in both sensitive and tolerant cultivars, indicating that global gene expression in tolerant and sensitive cultivars might be the same, but they are differently regulated in different cultivars. Further analysis confirmed our hypothesis. For instance, Potri.011G007800.1 (*WRKY6*) is upregulated in both sensitive and tolerant cultivars, whereas Potri.014G164300.1 (*WRKY1*) is upregulated in tolerant cultivars but downregulated in sensitive cultivars (Table S4). Similar results were also reported by Muthusamy et al. [48], who demonstrated that approximately 64 DEGs, which were upregulated in the tolerant cultivar were downregulated in the sensitive banana (*M. acuminata*) cultivar. These results show that plant responses to dehydration are highly complex, and significant cross-talk exists between these mechanisms at the molecular level.

Transcriptome-wide characterization of PopdaWRKYs showed that they are distributed all over the genome, with a maximum number of WRKY TFs on Chromosome 2, followed by Chromosome 1. A gene’s location on the chromosome plays a key role in determining the variation and evolution of a particular trait in an organism [40]. The high number of WRKYs found on Chromosome 2 might be because W-box elements often occur in clusters within the promoters [49]. Therefore, dehydration-responsive PopdaWRKYs may help to understand the adaptive mechanisms under water deficit conditions, and these chromosomes, which have a large number of clusters of W-box elements, might play a profound role in these adaptations. Mostly those genes that are present in the center of chromosomes are conserved, while those present on either end have a high chance of crossing-over and thus might be key players in adaptation and evolution processes. We also examined PopdaWRKY orthologs that were distributed throughout a range of species, from trees to even grasses (Table S2). This suggests that PopdaWRKYs are conserved throughout the plant kingdom, and that they may have similar functions across the species, too. This was further confirmed by the study of motif composition and by phylogenetic analysis of dehydration induced PopdaWRKYs and their orthologs in Arabidopsis and rice. Our results suggested that similar motif composition was found for a particular WRKY subgroup within the same group. Furthermore, they were also clustered together, indicating their phylogenetic similarity as well (Figure 6). In a previous study, salt-responsive members of WRKY TFs in cotton and their orthologs in other species were analyzed by Fan et al. [50], and they reported very similar results, confirming that all the studied species possess common motifs. Similarly, Jiang et al. [51] reported that phylogenetic analysis revealed that the member of WRKY family among poplars, Arabidopsis, and other species were divided into three groups and several subgroups based on the protein structure of WRKYs.

We further sought to determine the difference in protein sequences of dehydration-responsive and non-responsive WRKY TFs. The alignment of both types of WRKY TFs showed the presence of isoleucine (I) in dehydration-responsive WRKYs only, while the non-responsive did not have it (Figure S5). Literature reports suggest a regulatory role of isoleucine and the two branched chain amino acids leucine and valine by acting as precursors for a number of plant secondary metabolites involved in abiotic stress tolerance [52,53]. In another study, it was reported that isoleucine acts as a precursor for an important class of secondary metabolites glucosinolates. It is suggested that glucosinolates play an important role in plant development under altered environmental conditions.

GO terms for biological functions showed the highest fold enrichment for the regulation of transcription, followed by the regulation of RNA metabolic processes (Table 2), which may be due to the characteristic function of TFs, i.e., to regulate transcription. This was also confirmed by GO terms for molecular functions, which showed the highest fold enrichment for transcription factor activity, followed by nucleic acid binding activity (Table 2). This is because the heptapeptide sequence (WRKYGQK) in WRKY TFs specifically binds to consensus cis-acting elements (i.e., W-box, sequence = TTGACT/C) in the promoter region of target genes, regulating their transcription [28]. Significant fold enrichment was also found for nitrogen compound metabolic processes in GO terms for biological processes. This further elucidates the role of WRKY TFs in pathways involving one of the most important redox active molecules, the reactive nitrogen species (RNS). Previous reports have revealed the production of the RNS under abiotic stress conditions [54].

The KEGG pathway-enrichment analysis of dehydration-induced PopdaWRKYs showed that consecutive phosphorylation of mitogen-activated protein kinase kinase kinase1 (MAP3K1), mitogen-activated protein kinase kinase1 (MKK1), and mitogen-activated protein kinase4 (MPK4) leads to the activation of map kinase substrate 1 (MKS1), which in turn induces the expression of WRKY33. In a similar fashion but through a different route, consecutive phosphorylation of MAP3K1, MKK4, MPK3, and MPK6 indirectly induces the expression of WRKY22, which further interacts with FRK1, leading to early response to pathogen attack (Figure S3). The roles of dehydration-responsive PopdaWRKYs are both direct and indirect. The common receptor for bacterial flagellin, *flagellin sensitive2* (*FLS2*), activates the MAPK pathway that transfers extracellular and intracellular signals in plants in order to transmit information from sensors to responders [55]. The WRKY signaling cascade regulates both biotic and abiotic responses. Previous reports have established the fact that WRKY TFs modulate various stress responses through the mechanistic control of transcription. For instance, OsWRKY13 is reported to regulate the expression of more than 500 stress responsive genes [56]. Similarly, in one of the poplar species, *P. tomentosa*, overexpression of *PtoWRKY60* induced the expression of defense-related genes, pathogenesis-related protein 5.1 (PR5.1), PR5.2, PR5.4, PR5.5, and the constitutive expression of PR genes 5 (*CPR5*). Expression of *PtoWRKY60* was, in turn, induced by multiple signaling pathways such as jasmonic acid (JA), salicylic acid (SA), and salinity, and in response to insect (*Dothiorella gregaria*) attack [57]. Furthermore, *PtrWRKY73* from *P. trichocarpa* enhanced resistance to *Pseudomonas syringae* pv. tomato strain DC3000 (*Pst* DC3000) in Arabidopsis by inducing the expression of defense-related genes such as *PR1*, *PR2*, and *PAD4* [58].

We further validated RNA-seq results through qRT-PCR by selecting 14 different PopdaWRKYs representing members from Groups I, II, and III. A high correlation coefficient (R > 0.65) indicates high reliability of RNA-seq analysis. Diverse expression patterns were observed for different genes in different cultivars. For instance, *PopdaWRKY33* was upregulated in both sensitive and tolerant cultivars with the highest transcript accumulation compared to other members of the same group. The significance of WRKY33 in the MAPK pathway has already been mentioned in the above paragraph. Furthermore, *PopdaWRKY1* was upregulated in both tolerant cultivars and downregulated in Seogwang15 sensitive cultivars (Figure 7 and Figure S4). Previous reports have shown various roles for WRKY1 in other species. These include the regulation of stomatal movement in *A. thaliana* [59], the role in dehydration stress [60], and the enhancement of alkaloid production in opium [61].

**Figure 10:**
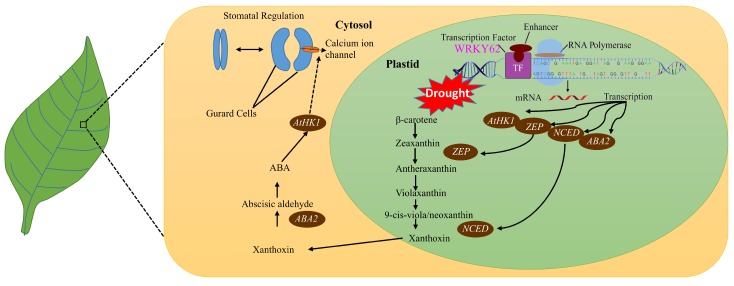
Model representing post-drought responses regulated by different genes: Three important genes *ZEP*, *NCED*, and *ABA2* controls important steps of ABA biosynthesis. The expression of these genes under dehydration conditions suggest that AtWRKY62 acts as enhancer transcription factor that induces the expression of ABA-biosynthesis related genes expression. AtHK1 which is downstream of ABA biosynthesis during drought condition was also downregulated in *atwrky62* KO lines (Figure 9C) suggesting that *AtWRKY62* may regulate *AtHK1* during dehydration response, which further regulates the movement of guard cells to modulate stomatal movement. This is also supported by Figure 8B, which shows the stomatal regulation after dehydration stress. The *atwrky62* plants were unable to close their stomata even after 30 m of dehydration stress.

In *atwrky62* plants, the basal expression of levels *ABA2*, *ZEP*, and *HK1* were found to be significantly low compared to WT plants, indicating a general suppression of the ABA pathway (Figure 9). This suppression was further reflected by the sensitive/wilted phenotype of these plants to dehydration caused by a consistent decline in transcript accumulation of the above-mentioned genes over time (Figure 8A). Our results indicating the possible role of Arabidopsis *AtWRKY62* in regulating drought stress tolerance through stomatal regulation is parallel to the findings of other studies as the Arabidopsis WRKY46 has been shown to regulate osmotic stress responses and stomatal movement independently [62]. Similarly, the cotton *WRKY41* and tomato (*Solanum pimpinellifolium* L3708) WRKY1 enhanced salt and drought tolerance by regulating somatal movement and ROS levels [63,64] with relevant changes in the expression patterns of the ABA pathway gene *NCED1*. Wheat (*Triticum aestivum*) WRKY1 and WRKY33 confer drought and heat stress tolerance in Arabidopsis and significantly alter the expression of the ABA pathway genes such as *ABA1*, *ABA2*, *ABI1*, *ABI5*, *DREB2b*, and *RD29A* [65]. Based on the functional genomics study and ABA biosynthesis related genes expression, this study suggests a putative model for WRKY TFs and their mode of action during dehydration conditions (Figure 10). We propose that WRKY62 act as transcription enhancer, inducing various dehydration related genes expression. This includes ABA biosynthesis-related gene expression such as *ZEP*, *NCED*, and *ABA2* (Figure 9A–D) that will help to accumulate more ABA in stressed plants. The production of ABA induces the expression of AtHK1 (Figure 9C), a known gene for stomatal regulation that regulates stomatal movement by modulating calcium channel activity [46]. Therefore, the inability to close stomata to prevent water loss in *atwrky62* KO line under dehydration conditions might be due to the reduced expression of AtHK1. Numerous reports have identified and reported the expression patterns of WRKY TFs from different species under various stress conditions. These include cotton [50,66,67], soybean [68], castor bean [69], strawberry [70], *Brassica napus* [71], banana [48], and Arabidopsis [72]. In a genome-wide survey of WRKY TFs in *P. trichocarpa* He and Dong [73] identified about 104 *P. trichocarpa* WRKYs (PtrWRKYs) and classified them into different groups. They also validated that PtoWRKY78 showed a significant induction to 0.1 mM SA or 25% PEG-6000 treatment. Similarly, in a recent study, Jiang and Duan [51] reported important information regarding WRKY TFs in *P. trichocarpa* and showed that 61 of the PtrWRKY genes were induced by biotic and abiotic treatments. However, the biological function of WRKY members in forest trees is still unknown. The current study is, to our knowledge, the first attempt toward identification of plant-specific WRKY TFs in *P. davidiana* through high-throughput RNA-seq analysis in response to dehydration-induced drought stress. This study will provide a platform for further studies in Populus and other forest trees.

## 4. Materials and Methods

### 4.1. Plant Material and Induction of Dehydration Stress

About 22 different *P. davidiana* (Korean Aspen) cultivars were screened for their tolerance or sensitivity toward dehydration stress, based on the development of symptoms. Six different cultivars, which included three sensitive (Seogwang15, Junguk6-2, and Palgong1) and three tolerant (Seogwang9, Palgong2, Odae19) cultivars, were selected and further confirmed by measuring ROS (hydrogen peroxide [H${}_{2}$O${}_{2}$]) accumulation through 3,3’-diaminobenzidine (DAB) staining. All the saplings of selected *P. davidiana* cultivars were obtained from the Korea Forest Research Institute (KFRI) and re-propagated on Murashige and Skoog (MS) medium (4.4 g MS, 3% sucrose, 0.27% gelrite, at a final pH 5.8, supplemented with 0.5 ppm naphthalene acetic acid [NAA]) by internode culture for the purpose of organogenesis, as described by Mun et al. [74], and kept at 23 ${}^{\circ }$C (16 h light/8 h dark cycle). Approximately six-week-old plants were used for dehydration stress treatment. In order to obtain a robust change in gene expression, plants were carefully uprooted from the medium, washed carefully with sterile distilled water, and left in open air for about 10 min to induce dehydration stress. The 10 min time point was selected based on an initial morphological screening and H${}_{2}$O${}_{2}$ accumulation. Immediately after the stress (0 min), there were no signs of wilting nor accumulation of H${}_{2}$O${}_{2}$ (Figure 1C), while at 20 and 30 min time points there was excessive accumulation of H${}_{2}$O${}_{2}$ (Figure 1C). Therefore, we selected 10 min for induction of dehydration stress to target DEGs that are involved during early responses to dehydration stress.

### 4.2. RNA Extraction and RNA Sequencing

RNA extraction and sequencing was done using the method described by Hussain et al. [75]. Briefly, RNA was extracted in triplicates from about eight weeks old *P. davidiana* leaf tissues using RNeasy^®^ Plant Mini Kit (Qiagen, Germantown, MD, USA), as per the manufacturer’s standard protocol provided with the kit. RNA integrity was analyzed using Agilent Bioanalyzer (Agilent, Santa Clara, CA, USA) and RNA libraries were generated using TruSeqTM RNA library prep kit (Illumina, San Diego, CA, USA). Subsequently, single stranded cDNA was synthesized using hexamer priming of mRNA, which was further used to synthesize double stranded cDNA libraries. These libraries were quantified using KAPA library quantification kit (Illumina, USA) and further processed for sequencing through HiSeq 2500 sequencer (Illumina, USA). The raw reads were processed to obtain high quality reads with a threshold level of Q20 > 40%. Reads with more than 10% ambiguous bases or with Q20 < 40% were removed, as suggested by Patel and Jain, [76]. These high quality reads were aligned against the *P. trichocarpa* genome using TopHat [77], keeping default parameters. The abundance of transcripts was determined using Cufflinks v2.2.1 [78], and differentially expressed genes (DEGs) were identified using Cuffdiff v2.2.1 [78]. The parameters used were as default values were geometric (–multi-read-correct –frag-bias-correct –max-mle-iterations 10,000-M transcripts.mask.gtf –emit-count-tables). Cuffdiff uses the test statistics T=E[log(y)]/Var[log(y)], where y is the ratio of the normalized counts between two conditions, and this ratio approximately follows a normal distribution; therefore, a *t*-test was used to calculate the P value for DEGs. The threshold level was p≤0.05. All the data generated through RNA-seq is available in public repository NCBI (https://www.ncbi.nlm.nih.gov/gds/?term=Drought+stress-mediated+transcriptome+profile+in+Populus+davidiana) under accession numbers GSE98175, GSE98174, GSE98173, GSE98172, GSE98171, and GSE98170 for all six varieties.

### 4.3. Identification and Classification of WRKY TFs in Transcriptome Data

The WRKY domain (PF03106) obtained from the PFAM protein family database (PFAM 31.0, March 2017) was used as query against the *P. trichocarpa* protein database v3.0 obtained from Phytozome (https://phytozome.jgi.doe.gov/pz/portal.html#). The use of *P. trichocarpa* as reference was because of close similarity with *P. davidiana* as to date the *P. davidana* genome was not published. For convenience, the *P. trichocarpa* gene accessions were used for *P. davidiana* WRKYs. The obtained gene accession numbers were used to identify *P. davidiana* WRKYs among the dehydration-responsive DEGs by applying conditional formatting in Microsoft Excel. A heatmap depicting hierarchical clustering and differences in expression values, and a multi-dimensional scattered (MDS) plot to measure dispersion in the data, were generated from the fragments per kilobase of transcripts per million mapped reads (FPKM) values in triplicates using R v3.3.1 (https://www.r-project.org/).

### 4.4. Identification of Common DEGs among Tolerant and Sensitive *P. davidiana* Cultivars

To study the common and/or unique DEGs shared by both sensitive and tolerant poplar cultivars, we compared dehydration-responsive DEGs from all *P. davidiana* cultivars, and analyzed them using online tool by VIB-UGENT (http://bioinformatics.psb.ugent.be/webtools/Venn/) keeping default parameters. The results obtained were presented in table form to show common and unique genes among different cultivars.

### 4.5. Chromosomal Location of *P. davidiana* WRKYs and Their Orthologs in Other Plant Species

To examine the location of poplar PopdaWRKY genes on chromosomes, the list of dehydration-responsive WRKY identifiers were queried against the *P. trichocarpa* genome using PhytoMine tool of Phytozome v12.0. The results, including gene names, length, chromosome number, and their positions on particular chromosomes, were exported in the form of a TSV file. The genes were then mapped to relative chromosomes and their positions on chromosomes were predicted using MapChart 2.32 with default parameters. To identify the orthologs of these PopdaWRKY TFs in other species of the plant kingdom, all the identified PopdaWRKY TFs were used as query against 68 different species in the PhytoMine tool of Phytozome v12.0, which searches each member of the provided list against every reference genome in its database.

### 4.6. Classification, Phylogenetic Analysis, and Motif Composition of PopdaWRKYs

Protein sequences of dehydration-responsive PopdaWRKY orthologs from *P. trichocarpa* were retrieved from Phytozome v12.0. All the sequences were aligned using ClustalW keeping default parameters (gap opening penalty 10, gap extension penalty for pairwise alignment 0.1, gap extension penalty 0.2, gap separation distance 4, and delay divergent cutoff 30%) in MEGA7 [44]. To identify conserved sites among all PopdaWRKYs orthologs, the conserved sequences were toggled at 90%. The WRKY motif was highlighted using the “Find motif sequence” option. Sequence identifiers were classified group-wise as Groups I, IIa, IIb, IIc, IId, IIe, and III. The phylogenetic relationships of dehydration-responsive. PopdaWRKYs and the presence of their orthologs in other species was evaluated by selecting 15 different PopdaWRKYs, i.e., five each from Groups I, II (1 from each subgroup), and III. The sequences of these PopdaWRKYs were queried against rice (*Oryza sativa*) and Arabidopsis (*Arabidopsis thaliana*) reference genomes using the Basic Local Alignment Search Tool for Proteins (BLASTP) available at the National Center for Biotechnology Information (NCBI) database (http://www.ncbi.nlm.nih.gov/), keeping default parameters. The proteins sequences thus obtained were aligned using the above-mentioned method, and the resultant alignment was used to generate a phylogenetic tree by the neighbor-joining method in MEGA7. The branching pattern of the tree was evaluated by running 1000 bootstrap replicates. To evaluate the structural divergence of WRKY TFs in poplar, rice, and Arabidopsis, the conservation of protein motifs was evaluated using the Multiple Expectation Maximization for Motif Elicitation program [79], keeping default parameters. To further evaluate the difference among dehydration-responsive and non-responsive WRKY TFs, the tolerant *P. davidiana* cultivars were screened for common genes and protein sequences of four common WRKYs having highest fold change were aligned with four non-responsive WRKYs using ClustalW with default parameters in MEGA 7.0. The alignment made was used to study differences between dehydration-responsive and non-responsive WRKY TFs.

### 4.7. Fold Enrichment Analysis

To further explore any possible biological role or molecular functions, the dehydration-responsive WRKYs were analyzed using the Gene Ontology (GO) consortium database (http://geneontology.org/), and their putative functions were searched using *P. trichocarpa* as a reference. GO terms for biological processes and molecular functions with *p* < 0.05 were selected and presented in Table 2.

### 4.8. KEGG Pathway Enrichment Analysis

To identify putative role of PopdaWRKYs in various metabolic pathways, the gene accession numbers of dehydration-responsive poplar DEGs were mapped to specific pathways using the “Search and Color Pathway” tool in the Kyoto Encyclopedia of Genes and Genomes (KEGG) (http://www.genome.jp/kegg/pathway.html) against the reference pathway. The dehydration-induced PopdaWRKYs involved in different pathways were colored red.

### 4.9. In Vivo Analysis of Stomatal Regulation by WRKY TF in Arabidopsis

To understand the role of WRKY TF genes in regulating dehydration stress tolerance in vivo, the protein sequence of PopdaWRKY62 (Potri.016G137900), which had the highest fold change in all tolerant cultivars (Table S4), was used as query against Arabidopsis genome. Based on the highest similarity *AtWRKY62* was selected for further in vivo analysis. As it is difficult to generate transgenic lines in Populus, we used the model plant Arabidopsis for further biological experiments using a reverse genetics approach. Arabidopsis WT (Col-0) and *atwrky62* mutant lines were ordered from the Arabidopsis Biological Resource Center [ABRC (https://abrc.osu.edu/)]. The knockout mutant line was genotyped to identify homozygous plants. WT and *atwrky62* plants were tested for its response to dehydration stress Seedlings of *atwrky62* and WT (Col-0) were sown on MS medium for four weeks. Seedlings were treated as described in Section 2.1, carefully up-rooted, and left in open air for 2, 5, 10, and 30 min, respectively. Stomatal structure was observed in WT and *atwrky62* by already described method [80]. Briefly, a thin layer of transparent nail polish was applied on the abaxial side of leaves and left to dry for an extended period of time. A transparent scotch tape was carefully applied on the dried nail polish and pressed gently to have an impression of the stomata. The tape was then mounted on a microscopic slide and studied under microscope (Axioplan 2 imaging, Carl Zeiss, Jena, Germany) at 400× magnification. The stomatal aperture of at least three stomata was then studied using the ruler tool in Adobe Photoshop CS6 and the pixel length was calculated for quantifying stomatal opening in WT and *atwrky62*.

### 4.10. Expression Analysis of ABA Biosynthesis and Signaling Genes

To examine if the stomatal closure is because of dehydration-induced ABA pathway, we selected some ABA biosynthesis and signaling-pathway-related genes (*ABA2, ZEP, NCED*, and *HK1*; Table S1). For this purpose, WT and *atwrky62* plants were exposed to dehydration stress as mentioned in Section 2.1, and samples were collected overtime (0, 2, 5, 10, and 30 min post dehydration stress). RNA extraction and qRT-PCR was performed as described earlier [81].

### 4.11. Validation through Real-Time Reverse Transcription PCR (qRT-PCR)

To validate dehydration induced transcriptional changes in the PopdaWRKYs obtained through RNA-seq mediated transcriptome analysis, we selected five PopdaWRKYs each form Groups I, II, and III, for qRT-PCR analysis. Details of these genes and their primer sequences are given in Table S1. RNA was extracted as mentioned in Section 2.2. cDNA was synthesized using BioFACT^TM^ RT Kit [(M-MLV, RNase H, BioFACT, Daejeon, Korea]. Briefly, 1 μg of RNA was mixed with 1 μL of Oligo (dT) primer (10 p mol) and incubated at 65 ${}^{\circ }$C for 5 min. A master mix containing 4 μL of 5× RT Reaction Buffer (containing mixed dNTPs) and 1 μL each of 8 mM dithiothreitol (DTT) and RTase (200 U/μL) was added to the preheated sample. RNase-free water was added to the reaction mixture to make up a final volume of 20 μL. The reaction mixture was incubated at 50 ${}^{\circ }$C for 1 h, followed by inactivation of RTase by incubation at 95 ${}^{\circ }$C for 5 min. PCR reaction was performed using cDNA as template in an Eco^TM^ Real-Time PCR machine (Illumina, USA), using 2× Real-Time PCR Master Mix (BioFACT, Korea) as per the manufacturer’s standard protocol. qRT-PCR was performed as per the method described by Imran et al. [81].

## Figures and Tables

**Table 1:** Common and unique PopdaWRKYs among different tolerant and sensitive cultivars.

Names	Total	Elements
Junguk62 Odae19 Palgong1 Palgong2 Seogwang15 Seogwang9	5	Potri.018G139300.1 Potri.016G128300.1 Potri.014G096200.1 Potri.006G263600.1 Potri.013G153400.1
Junguk62 Odae19 Palgong1 Palgong2 Seogwang9	1	Potri.004G060900.1
Junguk62 Odae19 Palgong2 Seogwang15 Seogwang9	5	Potri.006G105300.1 Potri.014G090300.1 Potri.003G182200.2 Potri.003G138600.1 Potri.018G019800.1
Junguk62 Odae19 Palgong1 Seogwang15 Seogwang9	1	Potri.004G007500.1
Junguk62 Odae19 Palgong1 Palgong2 Seogwang15	2	Potri.006G072400.1 Potri.001G044500.1
Junguk62 Odae19 Palgong2 Seogwang15	2	Potri.011G007800.1 Potri.006G109100.1
Junguk62 Palgong1 Seogwang15 Seogwang9	1	Potri.008G091900.1
Odae19 Palgong1 Seogwang9	1	Potri.008G103300.1
Odae19 Seogwang15 Seogwang9	1	Potri.014G024200.2
Junguk62 Odae19 Palgong2	3	Potri.007G047400.1 Potri.005G085200.1 Potri.016G137900.1
Odae19 Palgong1 Seogwang15	1	Potri.008G094000.1
Junguk62 Palgong1 Palgong2	1	Potri.006G133200.4
Odae19 Seogwang9	1	Potri.010G147700.1
Odae19 Palgong2	1	Potri.010G160100.3
Odae19 Palgong1	3	Potri.003G132700.1 Potri.002G168700.1 Potri.019G123500.1
Junguk62 Odae19	1	Potri.018G008500.1
Seogwang15 Seogwang9	1	Potri.010G163000.1
Junguk62 Seogwang15	1	Potri.005G141400.1
Odae19	1	Potri.014G111900.1
Seogwang9	4	Potri.002G059100.1 Potri.015G099200.1 Potri.005G203200.1 Potri.004G072000.1
Seogwang15	1	Potri.014G119800.1
Junguk62	1	Potri.005G055300.1 Potri.002G221600.1 Potri.016G083600.9

Note: The gene accession numbers from each cultivar were put in an online tool for making a Venn diagram (http://bioinformatics.psb.ugent.be/webtools/Venn/) using default parameters.

**Table 2:** Fold enrichment for GO terms of biological processes and molecular functions using the PANTHER overrepresentation test.

GO Terms	Reference List	*P. davidiana WRKYs*	Fold Enrichment	*p*-Value
		Biological Processes		
Regulation of transcription	2484	80	16.69	2.37 × 10${}^{-95}$
Regulation of RNA biosynthetic process	2524	80	16.43	8.50 × 10${}^{-95}$
Regulation of RNA metabolic process	2543	80	16.3	1.55 × 10${}^{-94}$
Macromolecule biosynthetic process	2607	80	15.9	1.13 × 10${}^{-93}$
Cellular biosynthetic process	2654	80	15.62	4.73 × 10${}^{-93}$
Regulation of biosynthetic process	2654	80	15.62	4.73 × 10${}^{-93}$
Nitrogen compound metabolic process	2681	80	15.47	1.06 × 10${}^{-92}$
Regulation of gene expression	2790	80	14.86	2.57 × 10${}^{-91}$
Regulation of cellular metabolic process	2881	80	14.39	3.36 × 10${}^{-90}$
macromolecular metabolic process	3071	80	13.5	5.56 × 10${}^{-88}$
Regulation of metabolic process	3103	80	13.36	1.27 × 10${}^{-87}$
Regulation of cellular process	5120	80	8.1	3.19 × 10${}^{-70}$
Regulation of biological process	5389	80	7.69	1.92 × 10${}^{-68}$
		**Molecular Function**		
Sequence-specific DNA binding	1057	80	39.23	4.01× 10${}^{-125}$
Transcription factor activity	1239	80	33.46	1.33 × 10${}^{-119}$
Nucleic acid binding activity	1239	80	33.46	1.33 × 10${}^{-119}$
DNA binding	2753	80	15.06	7.28 × 10${}^{-92}$
Heterocyclic compound binding	10024	80	4.14	5.77 × 10${}^{-47}$
Binding	14108	80	2.94	4.31 × 10${}^{-35}$
Molecular function	22374	80	1.85	4.54 × 10${}^{-19}$
Sequence-specific DNA binding	1057	80	39.23	4.01 × 10${}^{-125}$
Transcription factor activity	1239	80	33.46	1.33 × 10${}^{-119}$
Nucleic acid binding	4983	80	8.32	3.00 × 10${}^{-71}$

Note: Only GO terms with *p*-value < 0.05 are presented here. The detailed lists are given in Table S3.

## Supplementary Materials

The following are available online at http://www.mdpi.com/1422-0067/20/2/414/s1: Figure S1: Heatmaps showing expression patterns with dendrogram representing hierarchical clustering of both significant and non-significant DEGs in sensitive and tolerant cultivars. Figure S2: A multi-dimensional scattered (MDS) plot showing dispersion in data was made by FPKM values of drought induced WRKY DEGs using R. Figure S3: KEGG pathway analysis of drought-induced WRKY TFs. Figure S4: Validation of RNA-seq results using qRT-PCR in sensitive popular cultivars. Figure S5: Protein sequence alignment of dehydration-responsive and non-responsive WRKY TFs. Table S1: List of primers used in qRT-PCR validation of selected PopdaWRKYs their sequences and accession numbers. Table S2: Orthologs of PopdaWRKYs in other species. Table S3: GO terms for biological and molecular processes. Table S4: List of differentially expressed WRKYs and their fold change.

## Abbreviations

The following abbreviations are used in this manuscript:

ROS	reactive oxygen species
RNS	reactive nitrogen species
ABA	abscisic acid
TF	transcription factor
DEG	differential expressed gene
MDS	multi dimensional scatter plot
FPKM	fragment per kilobase million
GO	gene ontology
BLAST	basic local alignment search tool
KEGG	Kyoto Encyclopedia of Genes and Genomes
MAPK	mitogen-activated protein kinase
PAD3	phytoalexin deficient3
FLS2	flagellin sensitive2
PR5.1	pathogenesis-related protein5.1
SA	salicylic acid
*Pst*	*Pseudomonas syringae* pv. tomato
KO	knock out
KFRI	Korea Forest Research Institute
NAA	naphthalene acetic acid
MS	Murashige and Skoog
NCBI	National Center for Biotechnology Information.
