# Supplementary material for: WRKYs, the Jack-of-various-Trades, Modulate Dehydration Stress in Populus davidiana—A Transcriptomic Approach
Source: Int J Mol Sci. 2019 Jan 18;20(2):414. doi: 10.3390/ijms20020414 (PMC6358917; doi:10.3390/ijms20020414)
Supplement: Supplementary file 1 [file ijms-20-00414-s001.zip › Supplementary/Figure S1.docx]

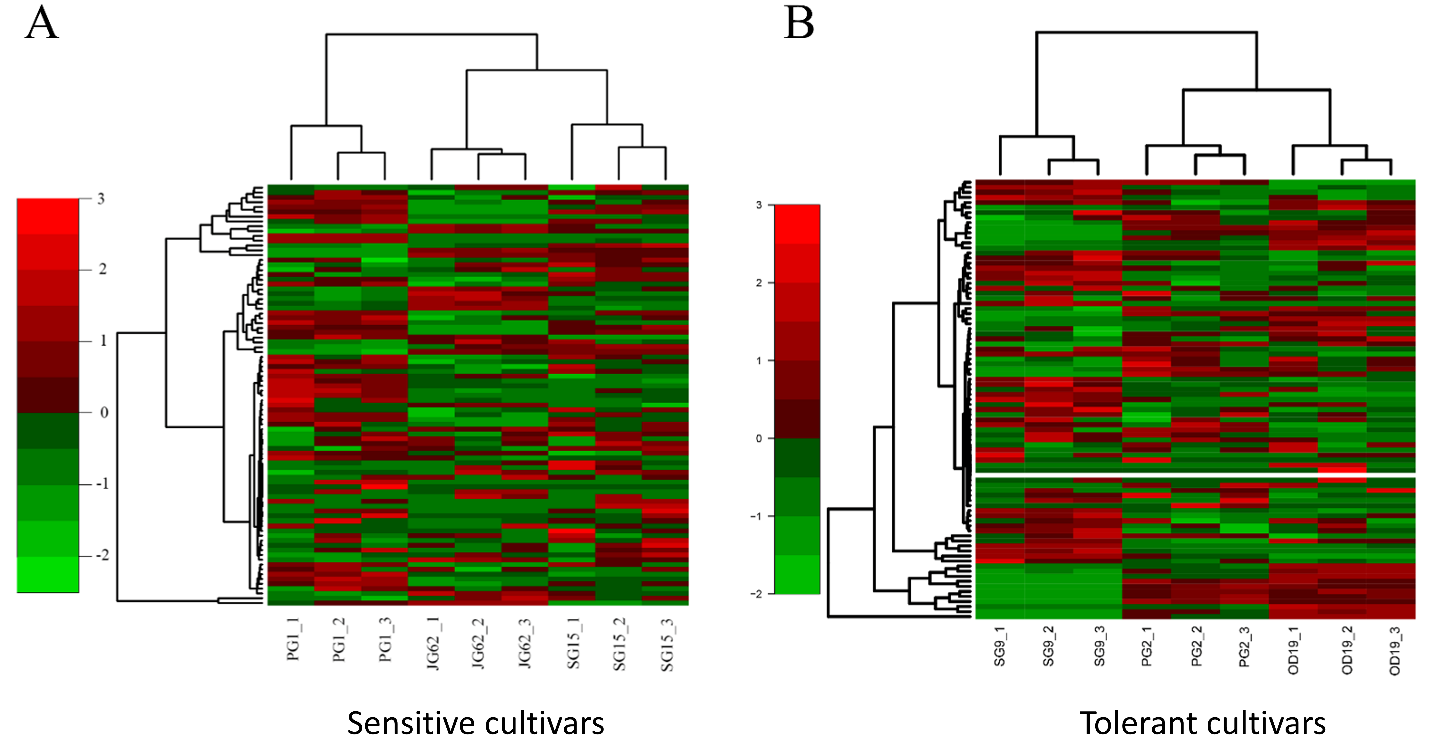


**Figure S1. Heatmaps showing expression patterns with dendrogram representing hierarchical clustering of both significant and non-significant DEGs in (A) sensitive and (B) Tolerant Cultivars**.
