# Supplementary material for: WRKYs, the Jack-of-various-Trades, Modulate Dehydration Stress in Populus davidiana—A Transcriptomic Approach
Source: Int J Mol Sci. 2019 Jan 18;20(2):414. doi: 10.3390/ijms20020414 (PMC6358917; doi:10.3390/ijms20020414)
Supplement: Supplementary file 1 [file ijms-20-00414-s001.zip › Supplementary/Figure S3.docx]

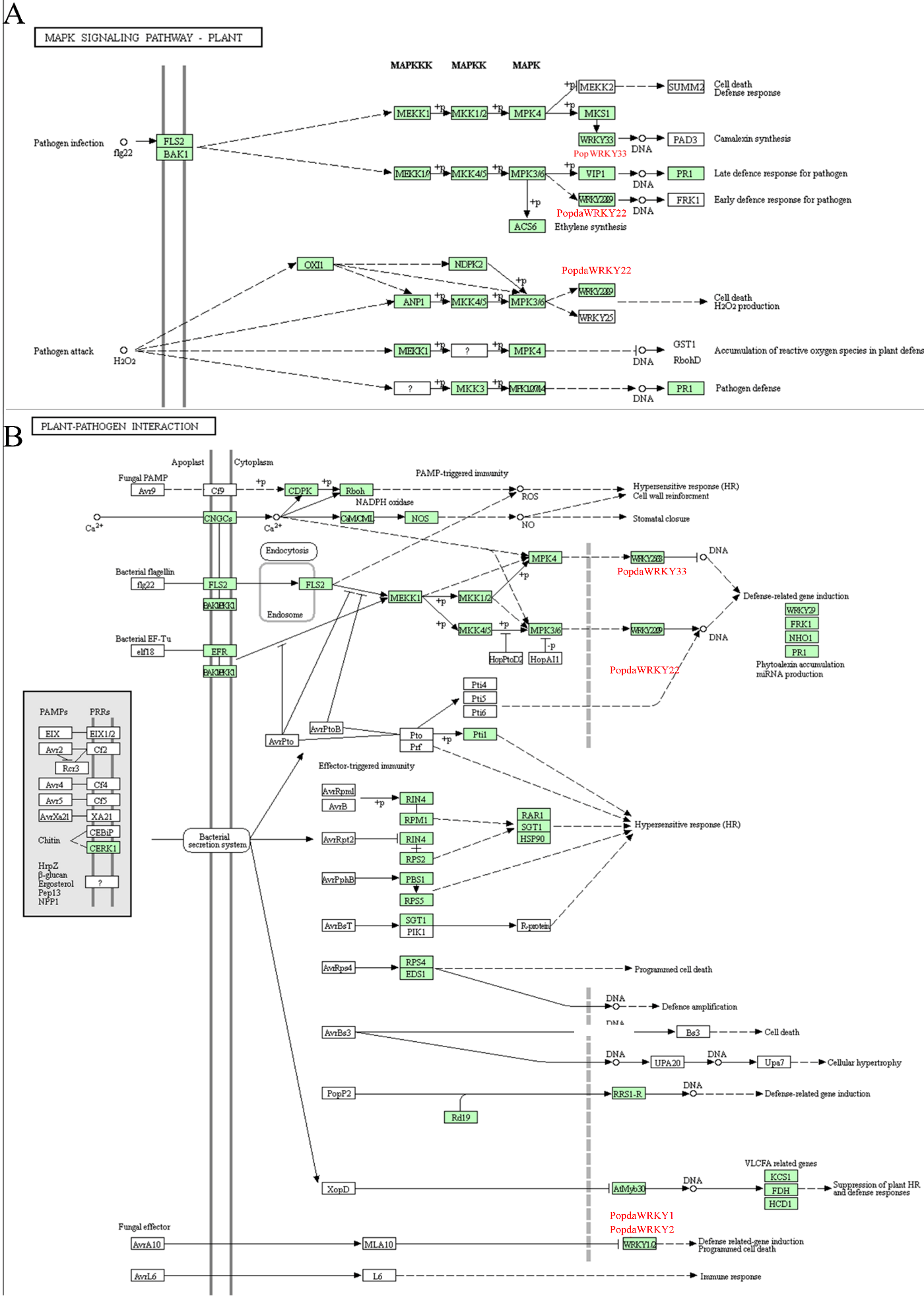


**Figure S3. KEGG pathway analysis of drought-induced WRKY** TFs. PopWRKYs were analyzed using the “Search and Color Pathway” tool in the Kyoto Encyclopedia of Genes and Genomes (KEGG) (http://www.genome.jp/kegg/pathway.html) against the reference pathway (KO). The red labels indicate drought-induced PopWRKY TFs.
