# Supplementary material for: WRKYs, the Jack-of-various-Trades, Modulate Dehydration Stress in Populus davidiana—A Transcriptomic Approach
Source: Int J Mol Sci. 2019 Jan 18;20(2):414. doi: 10.3390/ijms20020414 (PMC6358917; doi:10.3390/ijms20020414)
Supplement: Supplementary file 1 [file ijms-20-00414-s001.zip › Supplementary/Figure S4.docx]

**Figure S4.** Validation of RNA-seq results using qRT-PCR in sensitive popular cultivars. About fourteen different *PopdaWRKys* from Group I, II and III were analyzed. The qRT-PCR values were used to calculate log2 of fold change (white bars) in Seogwang15 (A, B and C) and Junguk6-2 (D, E and F) popular cultivars and compared with fold change from RNA-seq analysis (black bars). The *R* value represents correlation coefficient while error bars represents ± SE from at least three replicates.
