# Supplementary material for: WRKYs, the Jack-of-various-Trades, Modulate Dehydration Stress in Populus davidiana—A Transcriptomic Approach
Source: Int J Mol Sci. 2019 Jan 18;20(2):414. doi: 10.3390/ijms20020414 (PMC6358917; doi:10.3390/ijms20020414)
Supplement: Supplementary file 1 [file ijms-20-00414-s001.zip › Supplementary/Figure S5.docx]

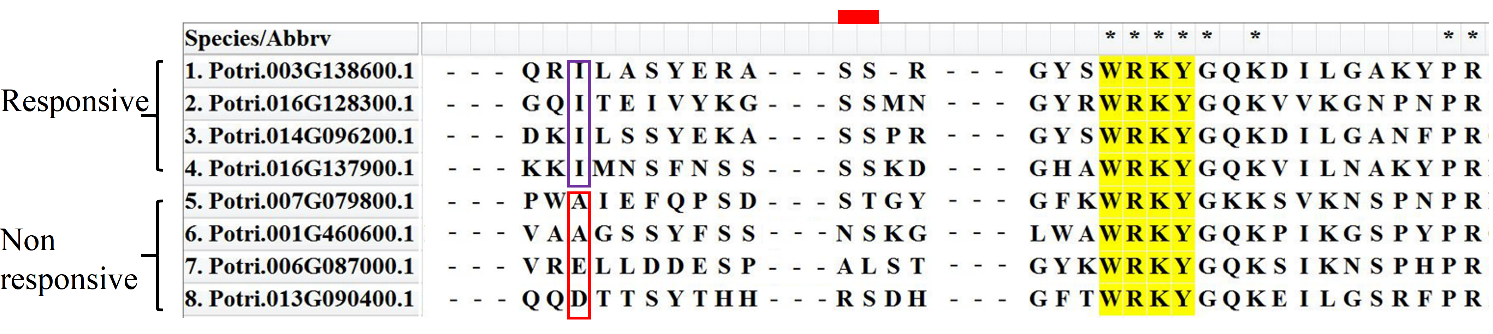


**Figure S5. Protein sequence alignment of dehydration-responsive and non-responsive WRKY TFs**. Four dehydration-induced WRKYs having highest fold change were selected among the common DEGs from tolerant cultivars and their protein sequences were aligned with four non-responsive WRKY TFs using ClustalW with default parameters in MEGA 7.0. The purple box represents the unique isoleucine (I) amino acids present only in dehydration responsive WRKYs. Red triangle represents serine (S) repeats. The complete protein sequences are not shown, dots represents missing protein sequences.
