# Supplementary material for: WRKYs, the Jack-of-various-Trades, Modulate Dehydration Stress in Populus davidiana—A Transcriptomic Approach
Source: Int J Mol Sci. 2019 Jan 18;20(2):414. doi: 10.3390/ijms20020414 (PMC6358917; doi:10.3390/ijms20020414)
Supplement: Supplementary file 1 [file ijms-20-00414-s001.zip › Supplementary/Table S1.docx]

Table S1: List of primers used in qRT-PCR validation of selected PopdaWRKYs their sequences and accession numbers.

| Gene Accession Number | Gene Name | Forward Primer (5’-3’) | Reverse Primer (5’-3’) |
| --- | --- | --- | --- |
| POPTR_0014s16220 | *PopdaWRKY1* | AACACCGCGGAAGGTCTCAC | CGGACAGGACGCAACCAACA |
| POPTR_0010s17040 | *PopdaWRKY4* | CGAGCTTGTCCATCGTCCCT | CTTCTGCTGCTGCTGGTGTG |
| POPTR_0001s37260 | *PopdaWRKY20* | GCGGTGCTAGGTACAAGCTG | ATCGTGATGCAAGCCGACCT |
| POPTR_0016s13600 | *PopdaWRKY33* | GGGCAGAAAGTCGTCAAGGG | GATTGGCCATGACGGAAGGC |
| POPTR_0016s08440 | *PopdaWRKY44* | GTTGAGGCTCGCCCTCAGTG | GCCCTGTCCCCGTTAGATGC |
| POPTR_0001s47670 | *PopdaWRKY2* | TAGGTTTGAGCGGCCTCCTG | ATGAGAAGCCAGGCGAAGGC |
| POPTR_0018s03450 | *PopdaWRKY11* | TCACGTTGCCGATTGCACTG | CGGCTGGTTGGGTTGAATCG |
| POPTR_0011s16050 | *PopdaWRKY14* | TGACAACGCTGGATCAGTGC | CCATGCCCACAAGTCAGATGGA |
| POPTR_0008s10280 | *PopdaWRKY48* | ACCACCACCACCACCAACAA | TGATGCCGGTGAAGGGACAA |
| POPTR_0021s00280 | *PopdaWRKY6* | CCCGTGTCCGAGGGCTTATT | TGCCATTGCAGCTGGAGGTA |
| POPTR_0002s06000 | *PopdaWRKY28* | ACCACGTATGAAGGGCAGCA | GGCTGTTGCTTGTGGATGAAGG |
| POPTR_0013s09460 | *PopdaWRKY55* | ACATTCGAGGTGGCGTACCG | ATCTGCTGCTGCTGGTGGAA |
| POPTR_0004s05920 | *PopdaWRKY65* | GAGGAGCAAGCTGGACCCAA | CGATGCCATGTCAGCATCCG |
| POPTR_0003s13250 | *PopdaWRKY22* | TGCCCATCCTACTCGAAGAAGC | CTGGCAAGGCAATTTCGTCCA |
| PopActin | *PopActin* | TTCTACAAGTGCTTTGATGGTGAGTTC | CTATTCGATACATAGAAGATCAGAATGTTC |
| AT2G17820 | *AtHK1* | GGCTGTTGTGGGGGTGACAA | TGAACAACGTGAGGGCGCTT |
| [AT5G01900](https://www.arabidopsis.org/servlets/TairObject?id=135602&type=locus) | *WRKY62* | TGGCCAAGTCCGTCCTCCAT | CTGGCATATGTGCTGGCCGA |
|  | NCED | TCCTCTGTTTCGTTCACGACG | CGTACGGAACCCTTGACGGA |
| AT5G67030 | ZEP/ABA1 | GCAATGGTATGCATTTCACG | TCATCGGCTTTGTCAGTGAG |
| [AT1G52340](https://www.arabidopsis.org/servlets/TairObject?id=28357&type=locus) | ABA2 | ACGGTTGATGATGTAGCGAACGCTGTT | CATCTGAAGACTTTAAAGGAGTGGTTAG |
| [AT5G57050](https://www.arabidopsis.org/servlets/TairObject?id=133380&type=locus) | ABI2 | GTTCTTGTTCTGGCGACGGAGC | CCATTAGTGACTCGACCATCAAG |
| AT3G18780 | *AtActin* | GCTGGACGTGACCTTACTGA | CCATCTCCTGCTCGTAGTCA |
