# Supplementary material for: WRKYs, the Jack-of-various-Trades, Modulate Dehydration Stress in Populus davidiana—A Transcriptomic Approach
Source: Int J Mol Sci. 2019 Jan 18;20(2):414. doi: 10.3390/ijms20020414 (PMC6358917; doi:10.3390/ijms20020414)
Supplement: Supplementary file 1 [file ijms-20-00414-s001.zip › Supplementary/Table S2.docx]

Table S2: Orthologs of PopdaWRKYs in other species

| **S. No** | **Organism** | **No of genes** |
| --- | --- | --- |
| **1** | *Manihot esculenta* | 83 |
| **2** | *Populus trichocarpa* | 83 |
| **3** | *Anacardium occidentale* | 82 |
| **4** | *Theobroma cacao* | 79 |
| **5** | *Prunus persica* | 78 |
| **6** | *Ricinus communis* | 76 |
| **7** | *Salix purpurea* | 76 |
| **8** | *Gossypium raimondii* | 75 |
| **9** | *Citrus clementina* | 74 |
| **10** | *Vitis vinifera* | 73 |
| **11** | *Eucalyptus grandis* | 72 |
| **12** | *Citrus sinensis* | 70 |
| **13** | *Malus domestica* | 70 |
| **14** | *Carica papaya* | 69 |
| **15** | *Glycine max* | 69 |
| **16** | *Phaseolus vulgaris* | 68 |
| **17** | *Eutrema salsugineum* | 67 |
| **18** | *Fragaria vesca* | 67 |
| **19** | *Arabidopsis thaliana* | 66 |
| **20** | *Medicago truncatula* | 66 |
| **21** | *Arabidopsis lyrata* | 65 |
| **22** | *Arabidopsis thaliana* | 65 |
| **23** | *Arabidopsis halleri* | 64 |
| **24** | *Boechera stricta* | 64 |
| **25** | *Capsella grandiflora* | 64 |
| **26** | *Brassica rapa FPsc* | 63 |
| **27** | *Capsella rubella* | 63 |
| **28** | *Linum usitatissimum* | 63 |
| **29** | *Panicum virgatum* | 63 |
| **30** | *Panicum virgatum* | 63 |
| **31** | *Kalanchoe laxiflora* | 62 |
| **32** | *Cucumis sativus* | 61 |
| **33** | *Kalanchoe fedtschenkoi* | 61 |
| **34** | *Setaria italica* | 61 |
| **35** | *Trifolium pratense* | 61 |
| **36** | *Ananas comosus* | 60 |
| **37** | *Brachypodium stacei* | 60 |
| **38** | *Chenopodium quinoa* | 60 |
| **39** | *Solanum lycopersicum* | 59 |
| **40** | *Musa acuminata* | 58 |
| **41** | *Setaria viridis* | 58 |
| **42** | *Brachypodium distachyon* | 57 |
| **43** | *Brassica oleracea capitata* | 57 |
| **44** | *Oryza sativa* | 57 |
| **45** | *Panicum hallii* | 57 |
| **46** | *Sorghum bicolor* | 57 |
| **47** | *Triticum aestivum* | 57 |
| **48** | *Zostera marina* | 57 |
| **49** | *Amborella trichopoda* | 55 |
| **50** | *Zea mays* | 55 |
| **51** | *Amaranthus hypochondriacus* | 54 |
| **52** | *Daucus carota* | 53 |
| **53** | *Mimulus guttatus* | 53 |
| **54** | *Solanum tuberosum* | 53 |
| **55** | *Spirodela polyrhiza* | 52 |
| **56** | *Oropetium thomaeum* | 48 |
| **57** | *Aquilegia coerulea* | 47 |
| **58** | *Physcomitrella patens* | 29 |
| **59** | *Sphagnum fallax* | 28 |
| **60** | *Selaginella moellendorffii* | 15 |
| **61** | *Marchantia polymorpha* | 14 |
| **62** | *Volvox carteri* | 14 |
| **63** | *Chlamydomonas reinhardtii* | 13 |
